# Efficacy and safety of docetaxel plus S-1-based therapy in gastric cancer: a quantitative evidence synthesis of randomized controlled trials

**DOI:** 10.3389/fphar.2023.1242548

**Published:** 2024-01-08

**Authors:** Hui-Fen Lv, Li-Feng Qin, Rui-Zhi Ran, Xue-Ping Jiang, Fang-Yu Zhao, Bo Li

**Affiliations:** ^1^ Hubei Provincial Key Laboratory of Occurrence and Intervention of Rheumatic Diseases, Hubei Minzu University, Enshi, China; ^2^ Department of Pharmacology, Health Science Center, Hubei Minzu University, Enshi, China; ^3^ Department of Gastroenterology, Minda Hospital of Hubei Minzu University, Enshi, China; ^4^ Department of Oncology, The Central Hospital of Enshi Tujia and Miao Autonomous Prefecture, Enshi, China; ^5^ Department of Pathology, Health Science Center, Hubei Minzu University, Enshi, China

**Keywords:** chemotherapy, DS-based regimen, meta-analysis, clinical outcome, adverse event

## Abstract

**Objective:** To systematically evaluate the safety and efficacy of docetaxel plus S-1-based therapy in gastric cancer treatment.

**Methods:** PubMed, Embase, The Cochrane Library, and Web of Science electronic databases were searched for randomized controlled trials on docetaxel plus S-1-based therapy in the treatment of gastric cancer from the establishment of the database to 1 September 2022. Relevant studies were included per pre-defined eligibility criteria, and two researchers independently screened and assessed the included literature using Review Manager v5. Outcome measures and statistics related with efficacy and safety profiles were extracted from the included studies, and Stata v15.1 was used for pooled analysis.

**Results:** Objective response rate (odds ratio = 2.34, 95% CI = [1.32, 4.13], *p* = 0.003), relapse-free survival (HR = 0.68, 95% CI = [0.58, 0.79], *p* < 0.001), progression-free survival (HR = 0.81, 95% CI = [0.68, 0.96], *p* = 0.016), and overall survival (HR = 0.86, 95% CI = [0.79, 0.95], *p* = 0.002) of docetaxel plus S-1-based therapy (DS-based therapy) in gastric cancer treatment were better than those of the non-DS-based therapy. However, DS-based therapy was associated with increased risk of certain adverse drug effects, such as alopecia, leukopenia, and oral mucositis. Further studies are warranted to validate the efficacy superiority of DS-based *versus* non-DS-based regimens as per our trial sequential analysis findings.

**Conclusion:** DS-based therapy significantly improves patients’ clinical outcomes in gastric cancer, albeit at the cost of increased toxicity. Further RCTs are needed to confirm the efficacy superiority of DS-based regimens.

## 1 Introduction

Gastric cancer (GC) is one of the most common malignancies, especially in Asian countries including China. GC ranks fifth and fourth in terms of incidence and mortality among all malignant cancer types (H Sung, et al., 2021). Surgical resection in combination with neoadjuvant/adjuvant chemotherapy, namely, multimodal therapy (Y Kakeji, et al., 2022), represents the mainstay of care for treatment of GC; however, tumor recurrence rates can reach 25%–40% in GC patients classified as stages II–IV (American Joint Committee on Cancers; AJCC2) (Bang, et al., 2012; Cristescu, et al., 2015; Kim, et al., 2005; Lee, et al., 2012; Macdonald, et al., 2001). Given the poor prognosis of advanced gastric cancer (AGC) patients, development of more effective chemotherapy regimens remains an unmet clinical need (Y Kurokawa, et al., 2021), which is also the current research hotspot (Zhu, et al., 2020). To date, fluoropyrimidine plus platinum compounds are the most widely used chemotherapeutic agents against GC worldwide (Cunningham, et al., 2008; Kang, et al., 2009; Koizumi, et al., 2008; Kurokawa, et al., 2021). S-1 monotherapy, which leverages the compounds tegafur, gimeracil, and oteracil potassium, is one of the most common treatments for stage II/III gastric cancer in Asia (Kakeji, et al., 2022; Sakuramoto, et al., 2007; Sasako, et al., 2011). However, as the cancer progresses to more advanced stages, S-1 monotherapy lacks efficacy and fails to reduce the incidence of hematogenous recurrence (Sakuramoto, et al., 2007; Sasako, et al., 2011). Recently, a long-term follow-up study of an international Phase III (START) trial showed that the docetaxel plus S-1 (DS) regimen significantly improved patients’ prognosis compared with S-1 monotherapy, although the initial analysis failed to show a significant difference in overall survival (OS) (Koizumi, et al., 2014). Although DS-based regimens may cause mildly increased toxicity compared with their non-DS-based counterparts, significantly improved efficacy outcomes were reported in multiple studies (Koizumi, et al., 2014; Kurokawa, et al., 2021). However, inconsistencies and discrepancies were observed among studies comparing DS-based therapy vs non-DS-based regimens in terms of efficacy and safety profiles (Kakeji, et al., 2022; Kang, et al., 2021; Kurokawa, et al., 2021; Lee, et al., 2019; Yamada, et al., 2019).

To the best of our knowledge, there is no systematic review/meta-analysis addressing this topic. Therefore, we by literature search identified randomized controlled trials (RCTs) on DS-based vs non-DS-based treatment regimens in gastric cancer and systematically assessed and quantitatively synthesized the relevant literature.

## 2 Materials and methods

### 2.1 Search strategy

The literature about the safety and efficacy of DS-based treatment for gastric cancer was searched in the database of PubMed, The Cochrane Library, Embase, and Web of Science from the establishment of the database to 1 September 2022. All RCTs comparing docetaxel plus S-1-based therapy with other treatments were considered. The language was limited to English. The following keywords were used to create the search strategy: “stomach neoplasms,” “docetaxel,” “S-1,” and “TS-1,” and all relevant MeSH terms of those words were considered as well. Those keywords were used in combination with logic “and” and “or” to ensure complete retrieval.

### 2.2 Inclusion criteria

The inclusion criteria are as follows: 1) subjects: patients aged ≥18 years with pathologically diagnosed gastric cancer; 2) interventions: DS-based regimens vs. non-DS-based controls; 3) efficacy outcomes: objective response rate (ORR), relapse-free survival (RFS), progression-free survival (PFS), and overall survival (OS); safety outcomes: adverse event rates, including hematologic toxicity and non-hematologic toxicity; 4) research type: RCTs; 5) language: English.

### 2.3 Exclusion criteria

The exclusion criteria are as follows 1) animal experiments; 2) the full text cannot be obtained; 3) data cannot be extracted; 4) meta-analysis, review, conference abstracts, and letters.

### 2.4 Risk of bias assessment

Risk of bias assessment for the included RCTs was performed as per standards recommended in Cochrane handbook (version 6.3). Evaluation criteria included randomization methods, concealed assignments, blinded participants, outcome evaluators, incomplete/missing outcome data, selective reporting, and other potential threats to validity. Quality assessment was conducted independently by two investigators (Rui-Zhi RAN and Xue-Ping JIANG) using the software RevMan 5, and discrepancies were resolved through discussion with a third reviewer.

### 2.5 Data extraction

Data were extracted independently by two reviewers (Hui-Fen LV and Li-Feng QIN). If there were any disagreements, a third reviewer was consulted, and differences were resolved through discussion. The extracted data included the following: baseline demographic characteristics, treatment regimens, efficacy outcome measurements, and detailed data on adverse events.

### 2.6 Statistical analysis

Stata v15.1 software was used for statistical analysis. Cochrane’s Q test *p*-value and *I* ^2^ statistic were used to analyze the heterogeneity of the included literature. In case that significant between-study heterogeneity was detected, as indicated by an *I* ^2^ ≥ 50% and/or Cochrane’s Q test *p*-value <0.1, the random-effects inverse-variance weighted meta-analysis model was used; otherwise, the fixed-effects model was used. Leave-one-out sensitivity analyses were performed to identify studies that contribute most to the heterogeneity. For dichotomous data, odds ratio (OR) and 95% confidence intervals (95% CI) were calculated based on event counts, as outcome measurements for meta-analyses. For time-to-event data, natural logarithmic hazard ratios and corresponding standard errors were extracted/calculated and pooled. For the efficacy outcomes, trial sequential analysis was performed using the *metacumbounds* command add-on to Stata, which was developed by Dr. Miladinovic and colleagues, to determine whether adequate information was accumulated for a definitive conclusion (Miladinovic, et al., 2013). The *a priori* diversity-adjusted information size (APDIS) was used as the information measurement. The pre-specified type I error was set as two-sided α = 0.05 and type II error as β = 20% (1-β = 80% power). A conservative relative risk reduction (RRR) of 15% was used as described previously (B Miladinovic, et al., 2013). O’Brian–Fleming boundaries were used (DeMets and Lan, 1994). Egger’s test was used to evaluate publication bias among the included studies, and a *p* > 0.05 of Egger’s test indicated no publication bias. A 95% CI not covering 1 or a *p* < 0.05 suggests statistical significance, unless otherwise specified.

### 2.7 Reporting guidelines

The present study was reported in line with the PRISMA (Preferred Reporting Items for Systematic Reviews and Meta-Analyses) reporting guidelines.

## 3 Results

### 3.1 Included literature and baseline characteristics

**FIGURE 1:**
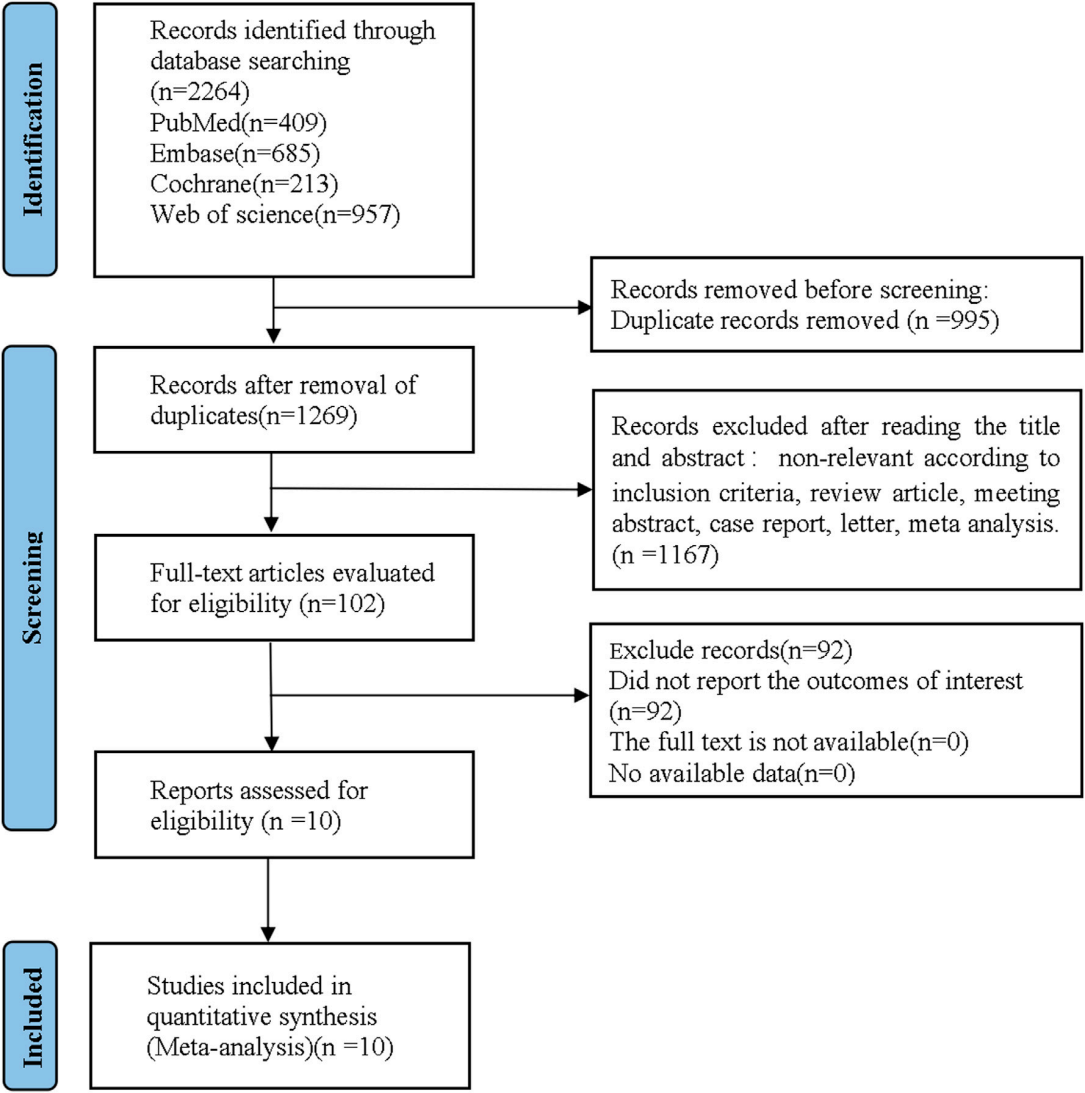
Flow diagram of the study as per Preferred Reporting Items for Systematic Reviews and Meta-analyses (PRISMA) style.

A total of 2,264 references were retrieved by literature search in electronic databases, and 10 RCTs were finally included for quantitative synthesis, after removing irrelevant literature according to inclusion and exclusion criteria. The flowchart of literature screening is shown in Figure 1.

A total of 10 trials were included in this meta-analysis, containing 4,145 patients. The details of baseline characteristics and types of participants are shown in Table 1 (Jeung et al., 2011; Koizumi, et al., 2014; Lee, et al., 2017; Lee, et al., 2019; Yamada, et al., 2019; Yoshida, et al., 2019; Zhu, et al., 2020; Kang, et al., 2021; Kurokawa, et al., 2021; Kakeji, et al., 2022).

**TABLE 1:** Baseline characteristics of included studies.

Study^a^	Year	Country	Sample size		Gender (M/F)	Disease stage	Age^b^ (years)		Intervention		Follow-up (months)	Outcome
			D	N			D	N	D	N		
1	2022	Japan	453	459	642/270	IIIA, IIIB, and IIIC	66	66	S-1: 80 mg (BSA< 1.25 m^2^); 100 mg (BSA: 1.25–1.5 m^2^); 120 mg (BSA≥1.5 m^2^); docetaxel (40 mg/m^2^)	S-1: 80 mg (BSA< 1.25 m^2^); 100 mg (BSA: 1.25–1.5 m^2^); 120 mg (BSA≥1.5 m^2^)	84	OS, RFS, and AEs
2	2021	Japan	30	30	38/22	Unresectable, recurrent	64	67	Docetaxel (40 mg/m^2^) + S-1: 40 mg (BSA< 1.25 m^2^); 50 mg (BSA: 1.25–1.5 m^2^); 60 mg (BSA≥1.5 m^2^)	Cisplatin (60 mg/m^2^)+S-1 S-1: 40 mg (BSA< 1.25 m^2^); 50 mg (BSA: 1.25–1.5 m^2^); 60 mg (BSA≥1.5 m^2^)	72	OS and PFS
3	2021	South Korea	238	246	384/158	IIA, IIB, IIIA, IIIB, and IIIC	58	58	CSC: docetaxel (50 mg/m^2^) + oxaliplatin (100 mg/m^2^) + S-1 (40 mg/m^2^) + surgery + S-1 (40–60 mg)	Surgery + S-1 (40–60 mg)	84	OS and PFS
4	2020	China	63	58	69/52	IIB, IIIA, IIIB, and IIIC	53.2	53.6	Docetaxel (75 mg/m^2^) + S-1 (40 mg/m^2^)	Oxaliplatin (130 mg/m^2^) + S-1 (40 mg/m^2^)	-	ORR
5	2019	Japan	454	459	643/270	IIIA, IIIB, and IIIC	66	66	Surgery + S-1: 80 mg (BSA< 1.25 m^2^); 100 mg (BSA: 1.25–1.5 m^2^); 120 mg (BSA≥1.5 m^2^);+ docetaxel (40 mg/m^2^)	Surgery + S-1: 80 mg (BSA<1.25 m^2^); 100 mg (BSA: 1.25–1.5 m^2^); 120 mg (BSA≥1.5 m^2^)	48	OS, RFS; and AEs
6	2019	Japan	370	371	513/228	Unresectable, recurrent	65	65	Docetaxel (40 mg/m^2^) + cisplatin (60 mg/m^2^) + S-1: 40 mg (BSA< 1.25 m^2^); 50 mg (BSA: 1.25–1.5 m^2^); 60 mg (BSA≥1.5 m^2^)	Cisplatin (60 mg/m^2^) +S-1: 40 mg (BSA< 1.25 m^2^); 50 mg (BSA: 1.25–1.5 m^2^); 60 mg (BSA≥1.5 m^2^)	66	OS, PFS, and AEs
7	2019	South Korea	75	78	102/51	IIIA, IIIB, and IIIC	54	58	S-1 (35 mg/m^2^) + docetaxel (35 mg/m^2^)	S-1 (35 mg/m^2^) + cisplatin (60 mg/m^2^)	72	OS, AEs,
												and DFS
8	2017	South Korea	DS	DC	34/12	-	55	55	Docetaxel (60 mg/m^2^) + S-1 (30 mg/m^2^)	Docetaxel (60 mg/m^2^) + cisplatin (60 mg/m^2^)	PFS, 20; OS, 40	OS, PFS, AEs, and ORR
			23	23								
9	2014	Japan	314	321	456/179	-	65	65	S-1: 80 mg (BSA< 1.25 m^2^); 100 mg (BSA: 1.25–1.5 m^2^); 120 mg (BSA≥1.5 m^2^) + docetaxel (40 mg/m^2^)	S-1: 80 mg (BSA< 1.25 m^2^); 100 mg (BSA: 1.25–1.5 m^2^); 120 mg (BSA≥1.5 m^2^)	60	OS, PFS, AEs
10	2011	South Korea	39	41	59/21	-	56	60	Docetaxel (35 mg/m^2^) + S-1 (35 mg/m^2^)	Docetaxel (35 mg/m^2^) + cisplatin (35 mg/m^2^)	37.5	ORR

^a^ Studies 1–10 refer to the following publications: (Jeung, et al., 2011; Koizumi, et al., 2014; Lee, et al., 2017; Lee, et al., 2019; Yamada, et al., 2019; Yoshida, et al., 2019; Zhu, et al., 2020; Kang, et al., 2021; Kurokawa, et al., 2021; Kakeji, et al., 2022), respectively.

^**b**^ Mean age.

Abbreviations: BSA, body surface area; D, DS-based therapy; N, non-DS-based therapy; OS, overall survival; PFS, progression-free survival; ORR, overall response rate; RFS, recurrence-free survival; AEs, adverse events.

### 3.2 Risk of bias profiles of included studies

**FIGURE 2:**
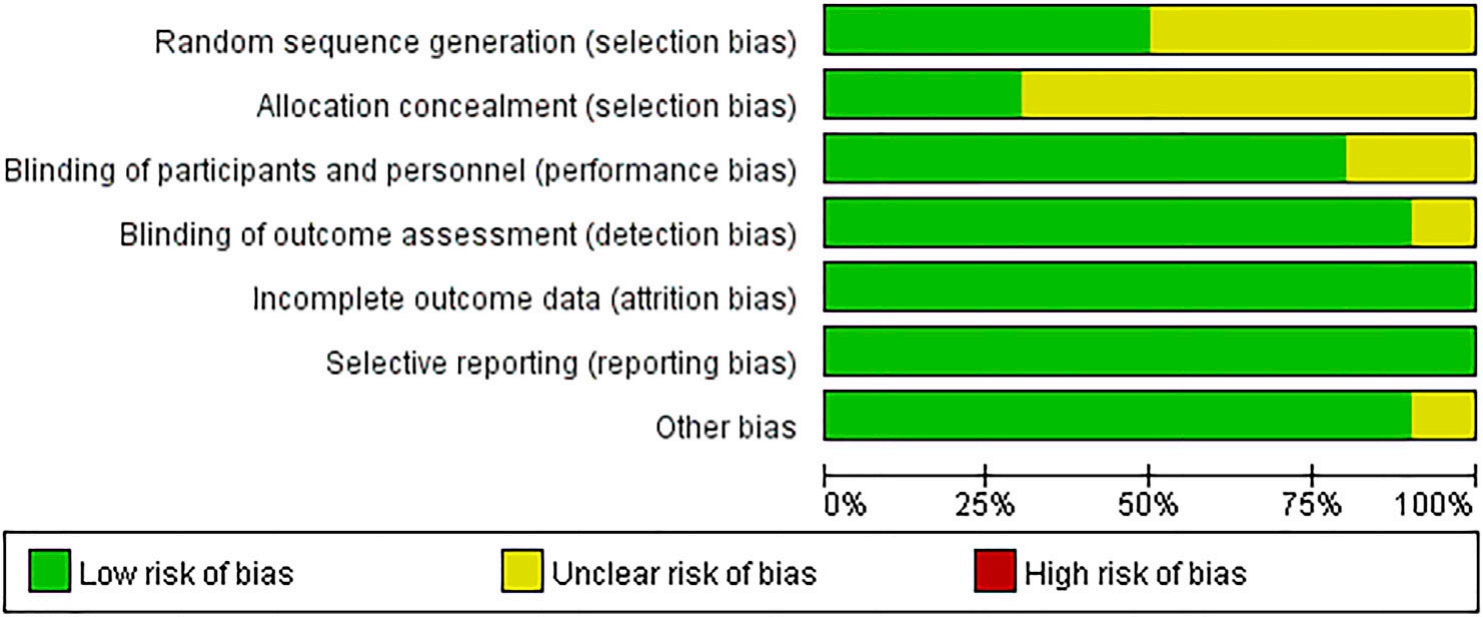
Risk of bias. Revman 5 software was used to evaluate the risk of bias. There is no high risk in assessment.

**FIGURE 3:**
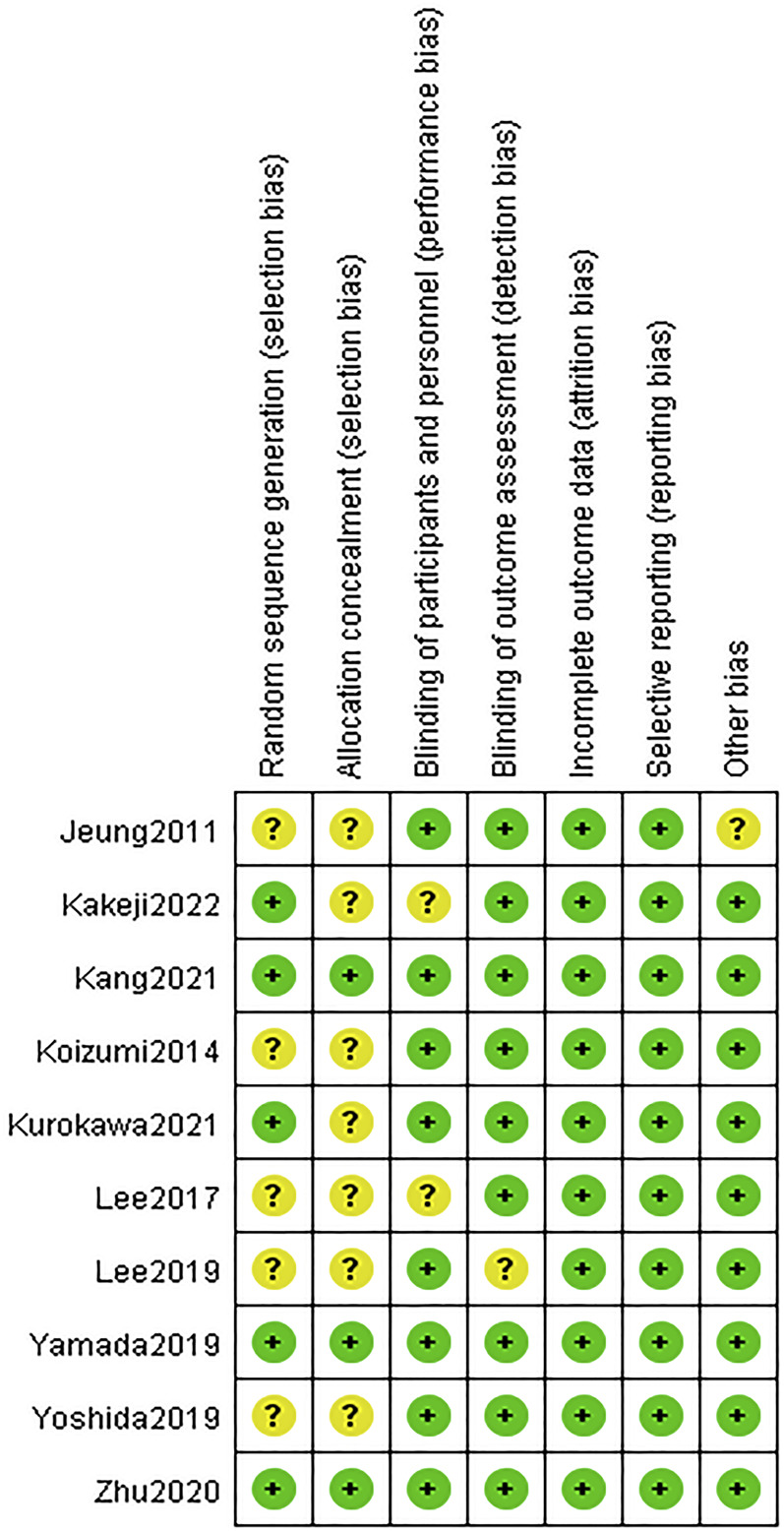
Risk of bias summary. Green indicates high quality. Yellow indicates that the article is unclear in this respect.

The included literatures were basically of high quality. There are uncertain risks in the generation of random sequences in five literatures and uncertain risks in the distribution in seven papers. The experimental results were objective data and were not affected by whether the distribution scheme was hidden or not. The details are listed in Figures 2, 3.

### 3.3 Efficacy profiles of DS-based vs. non-DS-based regimens

#### 3.3.1 Objective response rate (ORR)

**FIGURE 4:**
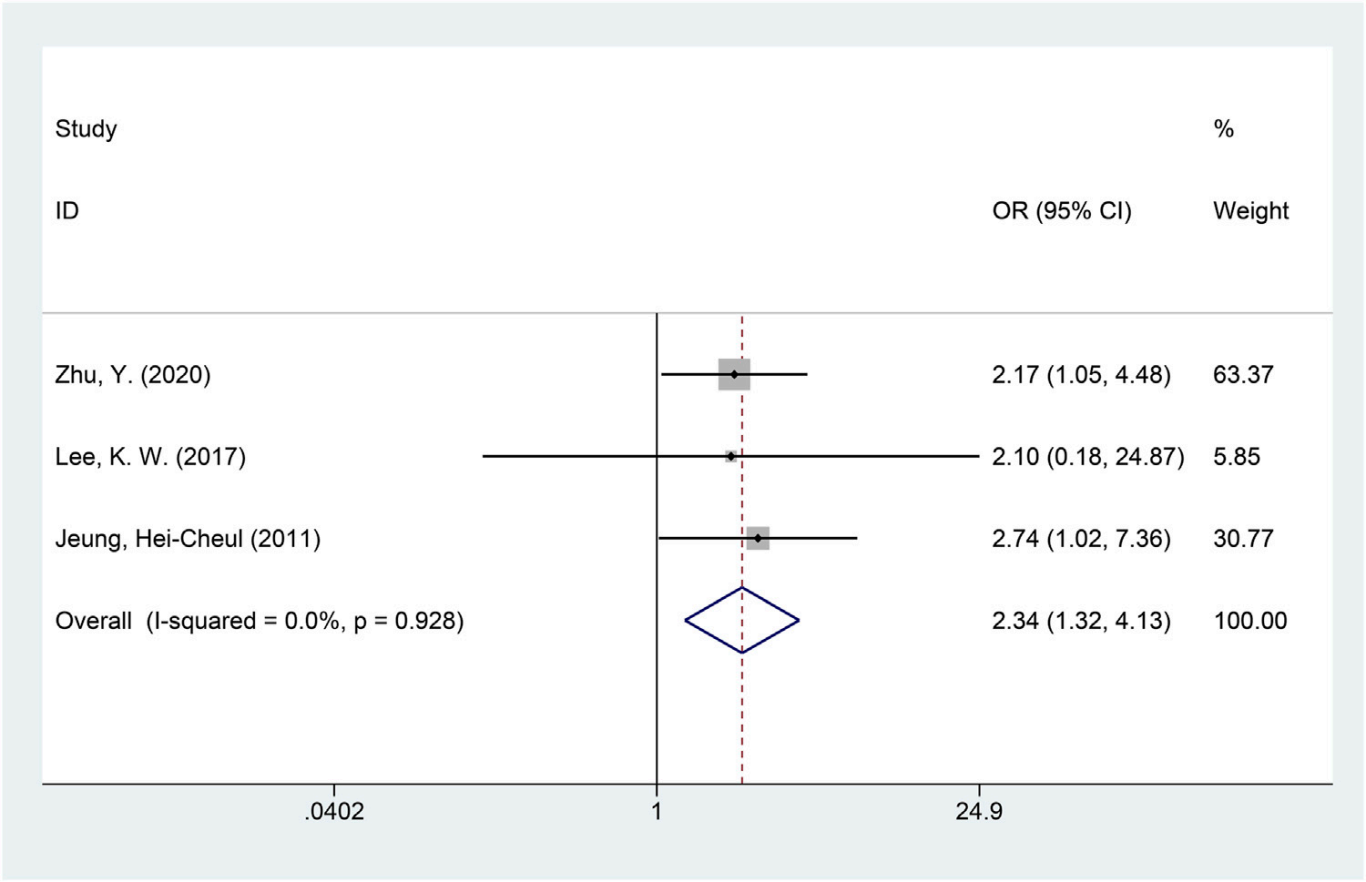
Forest plot of the objective response rate (ORR). The forest plot depicts the individual and pooled ORs with 95% CIs.

A total of three studies with available data on the ORR were included in the meta-analysis of the ORR (Jeung, et al., 2011; Lee, et al., 2017; Zhu, et al., 2020). The heterogeneity of the ORR among included studies was minimal (*I* ^2^ = 0.0%, *p* = 0.928), and the fixed-effects model was used for meta-analysis. The results of the analysis showed that the ORR of the DS-based therapy was better than that of the non-DS-based therapy (OR = 2.34, 95% CI = [1.32, 4.13], *p* = 0.003), and the difference was statistically significant. The meta-analysis forest plot is shown in Figure 4. Egger’s test suggested no publication bias (*p* = 0.942).

#### 3.3.2 Long-term survival

Only one RCT, the JACCRO GC-07, addressed the RFS (Kakeji, et al., 2022; Yoshida, et al., 2019), which reported superior RFS in the DS-based vs. the non-DS-based group (HR = 0.715; 95% CI = [0.59, 0.87]; *p* = 0.0008).

**FIGURE 5:**
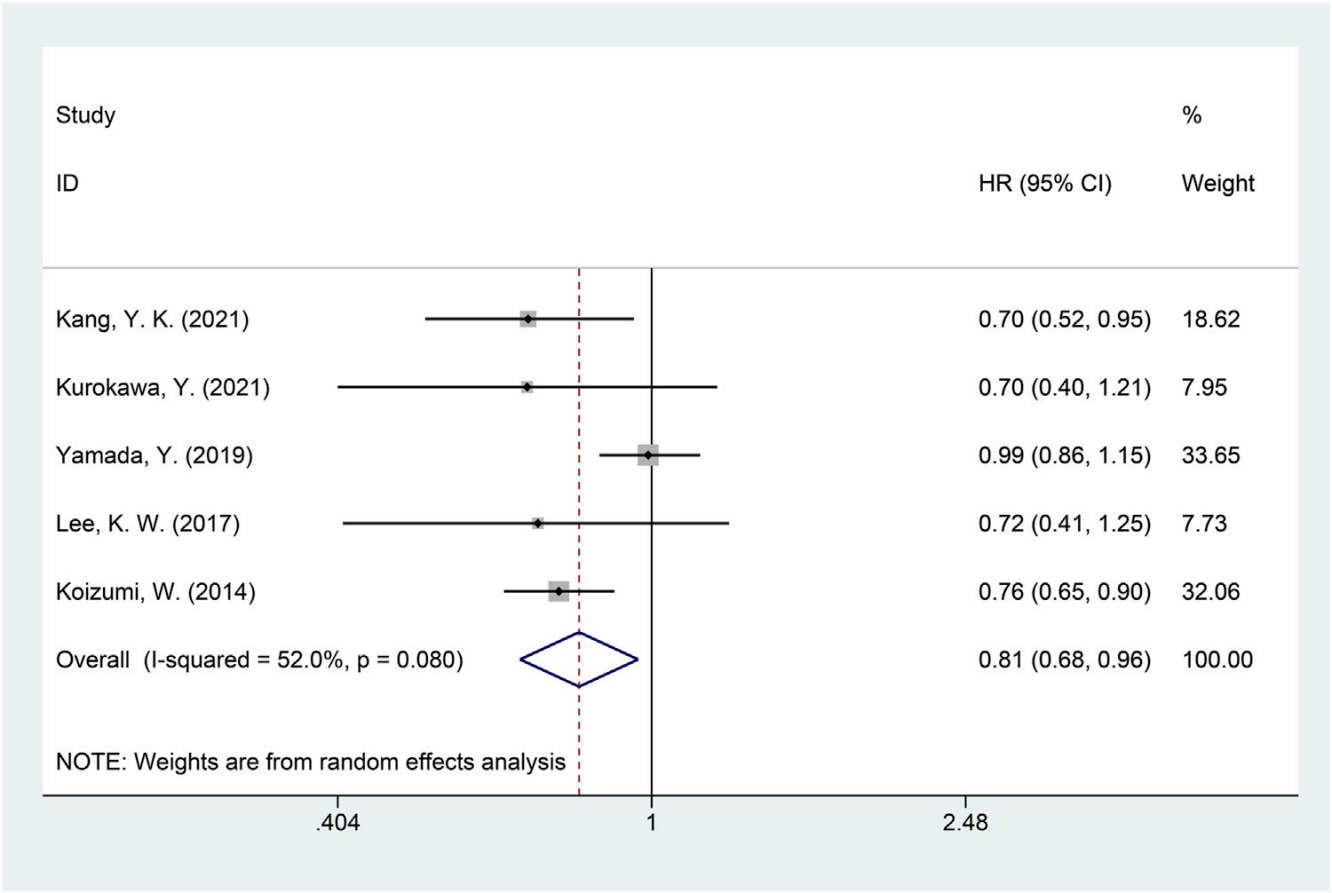
Forest plot of progression-free survival (PFS). The forest plot depicts the individual and pooled ORs with 95% CIs.

A total of five studies with available data on PFS were included in the meta-analysis of PFS (Kang, et al., 2021; Koizumi, et al., 2014; Kurokawa, et al., 2021; Lee, et al., 2017; Yamada, et al., 2019). Significant heterogeneity was detected (*I* ^2^ = 52.0%, *p* = 0.080), and the random-effects model was used for the meta-analysis of PFS. The results showed that, compared with the non-DS-based group, the DS-based group demonstrated significantly improved PFS (HR = 0.81, 95% CI = [0.68, 0.96], *p* = 0.016). The forest plot of meta-analysis is shown in Figure 5. Egger’s test suggested no publication bias (*p* = 0.335).

**FIGURE 6:**
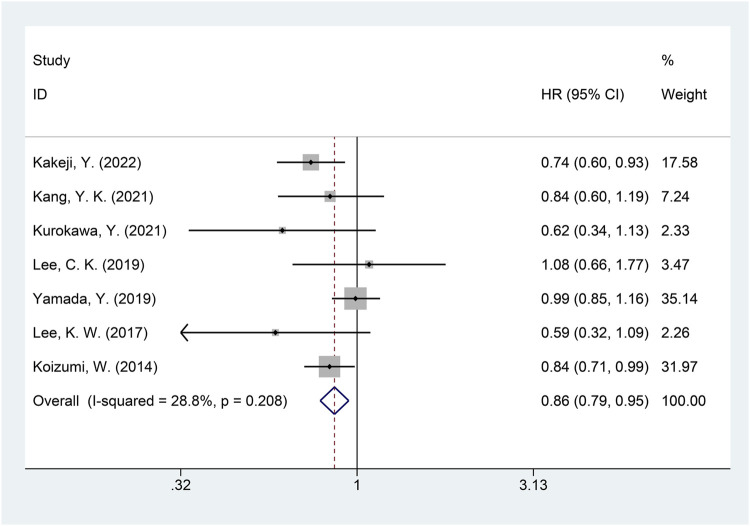
Forest plot of overall survival (OS). The forest plot depicts the individual and pooled ORs with 95% CIs.

A total of seven studies were included in the meta-analysis of OS (Kakeji, et al., 2022; Kang, et al., 2021; Koizumi, et al., 2014; Kurokawa, et al., 2021; Lee, et al., 2019; Lee, et al., 2017; Yamada, et al., 2019). The heterogeneity of OS among included studies was minimal (*I* ^2^ = 28.8%, *p* = 0.208), and the fixed-effects model was used for the meta-analysis of OS. The results showed that, compared with the non-DS-based group, the DS-based group demonstrated significantly improved OS (HR = 0.86, 95% CI = [0.79, 0.95], *p* = 0.002). The forest plot of OS is shown in Figure 6. Egger’s test suggested no publication bias (*p* = 0.313).

### 3.4 Safety profiles of DS-based vs. non-DS-based regimens

#### 3.4.1 Adverse events of all grades

The results of all-grade drug-induced adverse events suggested increased risk of developing alopecia (OR = 9.35, 95% CI = [1.94, 45.18], *p* = 0.005), oral mucositis (OR = 1.78, 95% CI = [1.47, 2.17], *p* < 0.001), and neutropenia (OR = 1.72, 95% CI = [1.21, 2.45], *p* = 0.002) in patients treated with DS-based regimens than in those treated with non-DS-based regimens. The heterogeneity of alopecia (*I* ^2^ = 96.3%, *p* < 0.001) and neutropenia (*I* ^2^ = 56.8%, *p* = 0.041) was high among studies, and the random-effects model was used for analysis. No single study with significant contribution to heterogeneity was found for alopecia and neutropenia. The studies on oral mucositis (*I* ^2^ = 49.0%, *p* = 0.098) had little heterogeneity and were analyzed by the fixed-effects model.

Except for the above adverse reactions, no statistically significant difference was observed between the DS-based and the non-DS-based groups in other all-grade adverse events. Detailed data of all grades of adverse events are listed in Table 2.

**TABLE 2:** Results of toxicity meta-analysis (all-grade adverse events).

Toxicity of all grades	#Trials	OR [95% CI]	*p*-value	Heterogeneity		Effect model	Egger’s test
				*I* ^2^ (%)	*p*-value		*p*-value
Alopecia	4	9.35 [1.94, 45.18]	0.005	96.3	0.000	R	0.594
ALT	3	1.00 [0.80, 1.25]	0.978	0.0	0.444	F	0.612
Anemia	5	1.14 [0.90, 1.44]	0.281	0.0	0.786	F	0.761
Anorexia	6	1.15 [0.81, 1.63]	0.435	59.4	0.031	R	0.976
AST	3	1.03 [0.83, 1.27]	0.813	11.7	0.322	F	0.640
Bilirubin increase	4	0.86 [0.48, 1.52]	0.596	64.6	0.037	R	0.124
Creatinine increase	4	0.48 [0.21, 1.11]	0.086	67.4	0.027	R	0.256
Diarrhea	6	1.16 [0.84, 1.59]	0.375	62.0	0.022	R	0.366
Fatigue	6	1.08 [0.90, 1.30]	0.387	31.3	0.201	F	0.921
Febrile neutropenia	4	2.63 [0.49, 14.29]	0.262	64.0	0.040	R	0.538
Leukopenia	4	1.48 [0.93, 2.37]	0.100	71.3	0.015	R	0.692
Oral mucositis	5	1.78 [1.47, 2.17]	0.000	49.0	0.098	F	0.875
Nausea	7	1.06 [0.89, 1.26]	0.490	40.1	0.124	F	0.238
Neutropenia	6	1.72 [1.21, 2.45]	0.002	56.8	0.041	R	0.674
Thrombocytopenia	5	0.77 [0.49, 1.20]	0.245	61.4	0.035	R	0.883
Vomiting	4	1.04 [0.84, 1.30]	0.702	0.0	0.881	F	0.983

Abbreviations: F, fixed-effects model; R, random-effects model; ALT, alanine aminotransferase; AST, aspartate aminotransferase.

#### 3.4.2 Adverse events of ≥ grade 3

The analysis of ≥ grade 3 drug-induced adverse events showed that leukopenia (OR = 3.04, 95% CI = [1.04, 8.94], *p* = 0.043), neutropenia (OR = 2.28, 95% CI = [1.36, 3.83], *p* = 0.002), and oral mucositis (OR = 2.07, 95% CI = [1.25, 3.44], *p* = 0.005) in the DS-based group were more frequent than in the non-DS-based group. The heterogeneity of leukopenia (*I* ^2^ = 89.2%, *p* < 0.001) and neutropenia (*I* ^2^ = 81.9%, *p* < 0.001) was significant, and the random-effects model was adopted for analysis. No single study with significant contribution to heterogeneity was found for leukopenia and neutropenia. The studies on oral mucositis had little heterogeneity (*I* ^2^ = 0.0%, *p* = 0.592), and the fixed-effects model was adopted for analysis.

Diarrhea (OR = 0.63, 95% CI = [0.45, 0.88], *p* = 0.007) was less common in the DS-based therapy than in the non-DS-based therapy. There was little heterogeneity among various studies on diarrhea (*I* ^2^ = 24.1%, *p* = 0.245), and the fixed-effects model was used for analysis.

There was no statistically significant difference between the DS-based therapy and the non-DS-based therapy in terms of other adverse events of ≥ grade 3. Detailed data are listed in Table 3.

**TABLE 3:** Results of toxicity meta-analysis (adverse events of ≥3 grade).

Toxicity ≥ grade 3	#Trials	OR (95% CI)	*p*-value	Heterogeneity		Effect model	Egger’s test
				*I* ^2^ (%)	*p*-value		*p*-value
ALT	4	1.16 [0.66, 2.04]	0.613	0.0	0.523	F	0.369
Anemia	5	0.93 [0.70, 1.25]	0.642	45.6	0.118	F	0.822
Anorexia	7	1.17 [0.95, 1.46]	0.142	0.0	0.519	F	0.019
AST	4	0.94 [0.55, 1.63]	0.839	0.0	0.458	F	0.659
Bilirubin increase	4	0.60 [0.29, 1.24]	0.169	19.9	0.290	F	0.076
Creatinine increase	5	0.68 [0.19, 2.42]	0.549	0.0	0.771	F	-
Diarrhea	7	0.63 [0.45, 0.88]	0.007	24.1	0.245	F	0.261
Fatigue	7	1.03 [0.74, 1.43]	0.860	0.0	0.781	F	0.864
Febrile neutropenia	5	3.35 [0.66, 16.99]	0.145	63.0	0.029	R	0.817
Leukopenia	5	3.04 [1.04, 8.94]	0.043	89.2	0.000	R	0.726
Nausea	8	1.31 [1.92, 1.87]	0.135	0.0	0.699	F	0.299
Neutropenia	7	2.28 [1.36, 3.83]	0.002	81.9	0.000	R	0.809
Oral mucositis	6	2.07 [1.25, 3.44]	0.005	0.0	0.592	F	0.697
Thrombocytopenia	6	0.76 [0.47, 1.21]	0.248	47.3	0.091	F	0.134
Vomiting	4	1.02 [0.58, 1.81]	0.946	0.0	0.827	F	0.861

Abbreviations: F, fixed-effects model; R, random-effects model; ALT, alanine aminotransferase; AST, aspartate aminotransferase.

### 3.5 Sensitivity analysis and publication bias

Egger’s test was conducted for assessing the publication bias of included studies. The results showed no bias in the ORR (*p* = 0.942), PFS (*p* = 0.335), and OS (*p* = 0.313). Bilirubin increase had a publication bias in all levels of adverse events. In ≥ grade 3 adverse events, anorexia and bilirubin increase were associated with publication bias. Among the adverse events, no bias was found in the analysis of other results, and the detailed *p* values are shown in Table 2 and Table 3. No studies that significantly influenced the results were found in the leave-one-out sensitivity analysis.

### 3.6 Trial sequential analysis (TSA)

**FIGURE 7:**
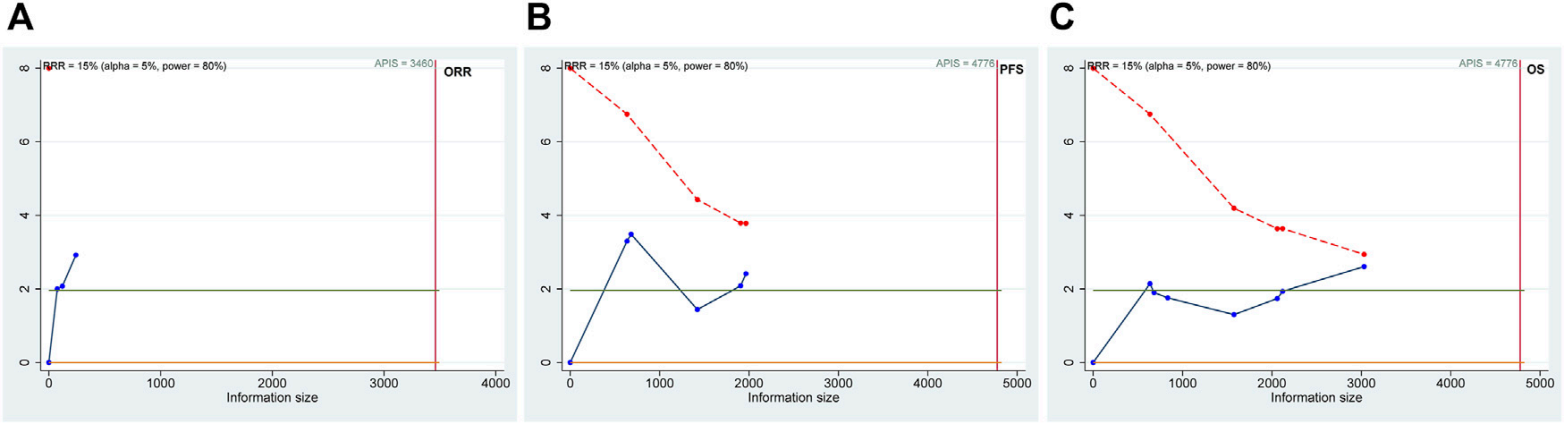
Trial sequential analysis (TSA) of the objective response rate (ORR; **(A)**), progression-free survival (PFS; **(B)**), and overall survival (OS; **(C)**) efficacy outcome measurements. The possibility of false positivity might exist in the ORR, PFS, and OS according to the results of TSA.

The results of TSAs are shown in Figure 7. For the ORR, PFS, and OS efficacy outcome measures, although the cumulative Z-curves passed the pre-specified α-threshold, none of them crossed the Lan–DeMets trial sequential monitoring boundaries, suggesting the possibility of false positivity. For RFS with only one trial available, we instead calculated the APDIS as per the formula (Z_α/2_+ Z_β_)^2^/[(1 − *w*) (1 − *S*)]×[(HR_0_ + 1)/(HR_0_–1)]^2^, as previously described, using the statistics based on a 3-year follow-up by Kakeji et al. (HR_0_ = 0.715, the censoring rate *w* was estimated as 0.5, and the average survival rate during the follow-up period was estimated as 0.5, as per the RFS Kaplan–Meier curve given in the original study) (Kakeji, et al., 2022; Miladinovic, et al., 2013). The estimated APDIS was 1,137, larger than the actually enrolled sample size, which was 912. Therefore, further studies plus updated meta-analysis are warranted to validate and confirm the currently observed efficacy effects of DS-based regimens.

## 4 Discussion

Our systematic review and meta-analysis on DS-based therapy vs. non-DS-based therapy shows the following: ORR, RFS (HR = 0.68, 95% CI = [0.58, 0.79], *p* < 0.001), PFS (HR = 0.81, 95% CI = [0.68, 0.96], *p* = 0.016), and OS (HR = 0.86, 95% CI = [0.79, 0.95], *p* = 0.002) in gastric cancer treatment, suggesting better efficacy profiles of DS-based therapy. However, DS-based therapy was associated with increased risk of certain adverse drug effects, such as alopecia, leukopenia, and oral mucositis.

Docetaxel is a paclitaxel drug, which can inhibit microtubule depolymerization. When used in combination with chemotherapy drugs (Van Cutsem, et al., 2006), docetaxel has been shown to increase the response rate in AGC patients (Zhu, et al., 2020). The anti-tumor mechanism of docetaxel is different from that of fluoropyrimidine and platinum, and there is no cross-resistance with these drugs. Docetaxel is often seen as a second-line regimen in the case of failed first-line therapy based on fluorouracil and/or platinum (Lee, et al., 2017). S-1 is a compound oral drug composed of tegafur, gimeracil, and oteracil potassium, which has a significant curative effect on advanced gastric cancer (Gyurkovska and Ivanovska, 2016). Fluorouracil phosphorylation could be blocked by oteracil potassium, so that the toxic effects of fluorouracil in the gastrointestinal tract could be reduced. Decomposition of fluorouracil could be inhibited by gimeracil, thus extending the action time of fluorouracil *in vivo*, maintaining blood drug concentration of fluorouracil, and ultimately preventing tumor growth (F Pietrantonio, et al., 2016). Docetaxel regulates the expression of enzymes involved in fluorouracil metabolism, which are thymidine synthase, dihydropyrimidine dehydrogenase, and rotary phosphotransferase, showing synergistic anti-tumor effects with fluorouracil, especially S-1 (Maeda, et al., 2004; Takahashi, et al., 2005; Wada, et al., 2006). Considering that the two drugs have a good synergistic effect in the anti-tumor mechanism, the survival rate of the DS-based therapy based on docetaxel plus S-1 is higher than that of the non-DS-based therapy.

We also analyzed adverse drug events, and the results showed that alopecia, oral mucositis, and neutropenia were more frequent in the DS-based therapy than in the non-DS-based therapy in all grades of adverse reactions. In grade 3 and higher adverse events, leukopenia, neutropenia, and oral mucositis were more common in the DS-based therapy than in the non-DS-based therapy. Diarrhea was less frequently observed in the DS-based therapy than in the non-DS-based therapy. Differences of adverse drug events between all above the DS-based therapy and the non-DS-based therapy were statistically significant.

In this analysis, neutropenia was more frequent for patients in the DS-based therapy group than those in the non-DS-based therapy in all grades of adverse events and ≥3 grade, which may be related to the fact that the main adverse event of docetaxel is myelosuppression. Despite the toxicity of docetaxel which may cause fatigue, anorexia, alopecia, leukopenia, and neutropenia (Koizumi, et al., 2014; Yoshida, et al., 2004; Yoshida, et al., 2006), all adverse events including febrile neutropenia (2%) were within the tolerable range in patients in our included studies, and no treatment-related deaths were reported (Fujitani, et al., 2014; Yoshida, et al., 2019). In the V325 study, for AGC patients approved to be treated with docetaxel plus cisplatin/5-fluorouracil in Western countries, the incidence rate of severe neutropenia was 82% (Van Cutsem, et al., 2006). Many modifications of docetaxel plus cisplatin/5-fluorouracil can be used with superiority in safety (Inal, et al., 2012; Koizumi, et al., 2014; Lorenzen, et al., 2007; Roth, et al., 2007; Tebbutt, et al., 2010). For management of grade 3 and higher hematologic adverse events, such as leukopenia and neutropenia, recombinant-human granulocyte colony-stimulating factor could be applied; therefore, these severe hematologic adverse events could be controlled and handled (Bian, et al., 2019).

Although meta-analysis found positive results of efficacy, for all efficacy outcome measurements considered in this work, the findings of TSA analysis suggest that the false positivity cannot be ruled out; thus, more original studies and updated meta-analysis are needed for further validation.

This study has certain limitations. First, the tumor stage was not analyzed as a potential confounding factor, and the efficacy and adverse effects were not analyzed by different stages of gastric cancer. This should be further explored in stage-stratified subgroup analyses when more data from additional RCTs become available. Second, the chemotherapy regimens used in the control groups of the included studies were heterogeneous, and different DS-based regimens were considered a common strategy while the possible differences were left unaddressed. Further head-to-head comparisons with increased number of trials and sample size, as well as network meta-analysis differentiating various treatment options, are warranted to validate our findings. Nevertheless, our study provides new insights for clinical management of gastric cancer.

## 5 Conclusion

DS-based therapy significantly improves the patients’ clinical outcome in gastric cancer, albeit at the cost of increased toxicity. Further RCTs are needed to confirm the efficacy superiority of DS-based regimens.

## Data Availability

The original contributions presented in the study are included in the article/Supplementary Material; further inquiries can be directed to the corresponding author.
